# Experimental infection of aquatic bird bornavirus in Muscovy ducks

**DOI:** 10.1038/s41598-022-20418-x

**Published:** 2022-09-30

**Authors:** Melanie Iverson, Alexander Leacy, Phuc H. Pham, Sunoh Che, Emily Brouwer, Eva Nagy, Brandon N. Lillie, Leonardo Susta

**Affiliations:** 1 Pathobiology, University of Guelph, Guelph, N1G2W1 Canada; 2 Animal Health Laboratory, University of Guelph, Guelph, N1G2W1 Canada

**Keywords:** Virus-host interactions, Viral infection

## Abstract

Aquatic bird bornavirus (ABBV-1), an avian bornavirus, has been reported in wild waterfowl from North America and Europe that presented with neurological signs and inflammation of the central and peripheral nervous systems. The potential of ABBV-1to infect and cause lesions in commercial waterfowl species is unknown. The aim of this study was to determine the ability of ABBV-1 to infect and cause disease in day-old Muscovy ducks (n = 174), selected as a representative domestic waterfowl. Ducklings became infected with ABBV-1 through both intracranial and intramuscular, but not oral, infection routes. Upon intramuscular infection, the virus spread centripetally to the central nervous system (brain and spinal cord), while intracranial infection led to virus spread to the spinal cord, kidneys, proventriculus, and gonads (centrifugal spread). Infected birds developed both encephalitis and myelitis by 4 weeks post infection (wpi), which progressively subsided by 8 and 12 wpi. Despite development of microscopic lesions, clinical signs were not observed. Only five birds had choanal and/or cloacal swabs positive for ABBV-1, suggesting a low potential of Muscovy ducks to shed the virus. This is the first study to document the pathogenesis of ABBV-1 in poultry species, and confirms the ability of ABBV-1 to infect commercial waterfowl.

## Introduction

Since the early twentieth century, Borna disease virus 1 (BoDV-1) has been recognized as the causative agent of Borna disease, a chronic neurological disease of horses, sheep, and rodents in central Europe, named after the city of Borna in Saxony, Germany^1^. Classified in the *Bornaviridae* family within the *Mononegavirales* order, BoDV-1 was the only virus in this family until 2008, when the novel parrot bornaviruses (PaBVs) were shown to be the causative agent of proventricular dilatation disease (PDD) in psittacines^2–5^. This disease affects the nervous system and is characterized by peripheral polyneuritis and in some cases meningoencephalitis^6^. Birds with PDD typically show gastrointestinal signs including passing of undigested seeds in feces, regurgitation, and dilation of the proventriculus due to loss of intestinal tone leading to weight loss and poor body condition. In some cases, neurological signs, including ataxia, paralysis, and seizures are seen^6,7^. Through a retrospective study of postmortem submissions of wild Canada geese and swans from Southern Ontario, in 2011 researchers from the University of Guelph identified a new bornavirus, later named aquatic bird bornavirus (ABBV-1)^8,9^. This virus appeared associated with lymphoplasmacytic inflammation of the central and peripheral nervous systems, as well as gross pathological features similar to those reported for PDD in psittacine birds^9,10^.

Discovery of these new viruses, among others, led to the reorganization of the *Bornaviridae* family into 3 genera encompassing 11 species, with the *Waterbird 1 orthobornavirus* divided into aquatic bird bornavirus 1 and aquatic bird bornavirus 2 (ABBV-2). ABBV-1 is most commonly identified in Canada geese and swans, and ABBV-2 has been detected in North American ducks (mallards and a wood ducks) exclusively^2,10^.

Since its original description, ABBV-1 has been detected or isolated from multiple species of waterfowl^11,12^, both in asymptomatic and neurologically affected birds, and sporadically in birds outside the Anseriformes order, including gulls^13^, bald eagles^6^, and an emu^14^. In fact, epidemiological studies have shown that wild waterfowl species appear to be the largest reservoir of ABBV-1 in North America and Europe, with an estimated prevalence of up to 50%, in some populations depending on species, flock, and location^15–20^. Aquatic bird bornaviruses, therefore, could be considered as primarily waterfowl viruses that may sporadically infect or become established in new avian populations^6^, as also supported by phylogenetic analysis, which suggests that horizontal spread is a driving force of interspecies transmission of bornaviruses in avian species^19^.

Experimental infection and/or PDD-like disease have been successfully reproduced by inoculating PaBVs into cockatiels^21–29^ and conures^30^, and canary bornaviruses (CaBVs) into canaries^29,31^. To date, however, there is no data regarding the ability of ABBV to experimentally infect waterfowl species. Therefore, the goal of the present study was to document experimental infection of ABBV-1 in the Muscovy duck, selected as a representative waterfowl, and to assess the suitability of this species to serve as an animal model of ABBV-1 infection.

## Results

### Clinical disease and gross findings

Groups of Muscovy ducks were inoculated either intracranially (IC), intramuscularly (IM) or orally (*per os*, PO) with ABBV-1. Ducks in the control (CO) group were sham-infected with carrier only through all 3 routes (Supplementary Figure S1). Throughout the course of the experiment (12 weeks), 4 ducks were found dead. One duckling in each of the IC and CO group died immediately after intracranial inoculation due to peracute cerebral hemorrhage (excluded from the study). One duck in the IM and one in the PO inoculation group were found dead at 3 and 46 days post inoculation (dpi), respectively, with no prior signs of illness and no evidence of gross or histological lesions. No clinical signs or additional unexpected deaths were observed during the entire course of the study across any of the experimental groups.

### Microscopic findings

#### Frequency and semi-quantitative scoring of nervous lesions

**Figure 1:**
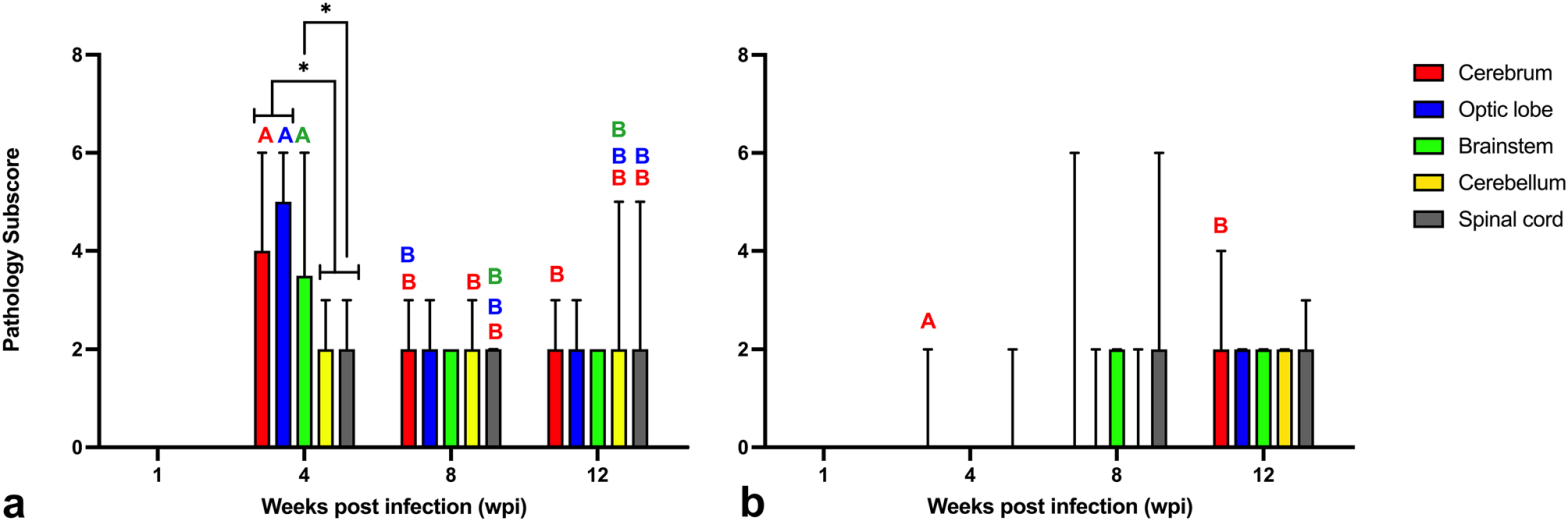
Graphs showing the pathology inflammation subscores in different areas of the brain of Muscovy ducks experimentally infected with ABBV-1 through the intracranial (**a**) or intramuscular (**b**) routes of infection, and assessed at 1, 4, 8, and 12 weeks post infection (wpi). Significant differences between terms *within* the same time point are identified with simple binary connectors. Flat lines at the end of a connector indicate differences with multiple underlying terms (significance level is reported for the highest p value among the comparisons). Significant differences of single organs at 4 wpi compared to organs at other time points are identified by different letters and color-coded (brain, red; optic lobe, blue; brainstem, green). Pairwise comparisons with data points scoring 0 (no inflammation) are not represented. Data columns represent median with data range. Krurskal-Wallis test with Benjamini, Krieger, and Yekutieli procedure for false discovery rate (* < 0.05, ** < 0.01, *** < 0.001).

**Figure 2:**
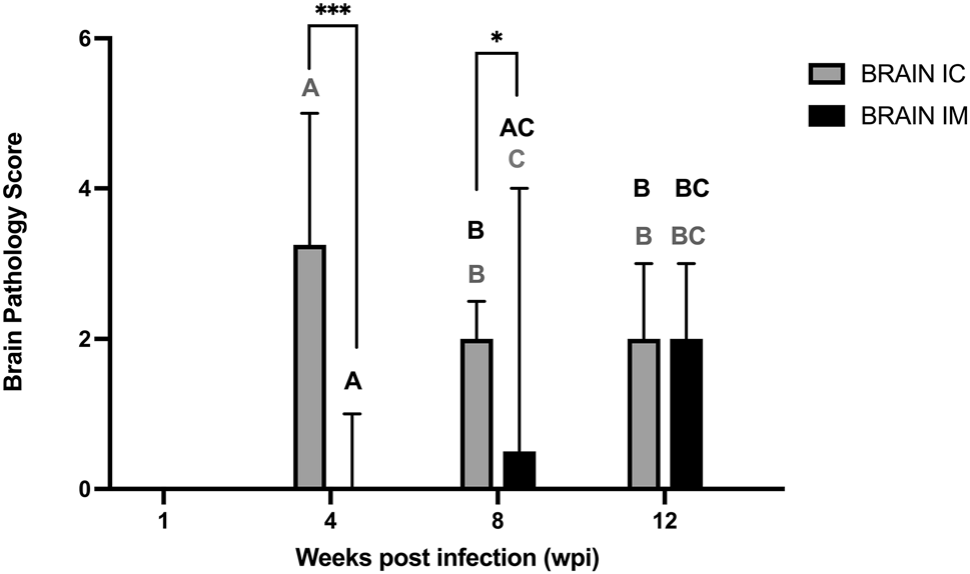
Graph showing the overall brain scores of inflammation in Muscovy ducks experimentally infected with aquatic bird bornavirus 1 (ABBV-1) through the intracranial (IC) or intramuscular (IM) routes of infection, and assessed at 1, 4, 8, and 12 weeks post infection (wpi). Significant differences between terms *within* the same time point are identified with simple binary connectors. Significant differences of terms at 4 wpi and terms at other time points are identified by different letters and color-coded (IC, grey; IM, black). Pairwise comparisons with data points scoring 0 (no inflammation) are not represented. Data columns represent median with data range. Krurskal-Wallis test with Benjamini, Krieger, and Yekutieli procedure for false discovery rate (* < 0.05, ** < 0.01, *** < 0.001).

Forty-eight ducks underwent detailed pathological assessment (3 birds/group/time point). Microscopic lesions were not identified in the PO or CO ducks, and lesions attributable to ABBV-1 infection in the nervous system (i.e., perivascular lymphocytic cuffs) were identified exclusively in the IC and IM ducks. In order to increase the definition of the histopathological assessment, the brain, spinal cord, proventriculus, ventriculus, and gonads from an additional 22 IM and 26 IC ducks were also evaluated (Supplementary Figure S1). The frequency and severity of microscopic lesions in the nervous system of the IC and IM groups is reported in Table 1 and Figs. 1 and 2; no additional microscopic changes or background lesions were observed throughout the course of the study.

**Table 1:** Frequency of inflammation and gliosis in different areas of the nervous system of Muscovy ducks inoculated with ABBV-1 by the intracranial (IC) and intramuscular (IM) routes at 1, 4, 8, and 12 weeks post infection (wpi).

Affected area or disease category	IC				IM			
	1 wpi	4 wpi	8 wpi	12 wpi	1 wpi	4 wpi	8 wpi	12 wpi
Cerebrum^a^	0/3^d^	10/10	10/10	15/15	0/3	1/10	4/10	10/11
Optic lobe	0/3	3/3	3/3	3/3	0/3	0/3	1/3	2/3
Brainstem	0/3	4/4	2/2	3/3	0/3	0/3	2/3	2/3
Cerebellum	0/3	7/8	7/7	9/12	0/3	0/10	3/7	6/9
Spinal cord	0/3	7/10	7/10	10/13	0/3	3/10	6/8	7/11
Brain meninges	0/3	10/10	9/10	14/14	0/3	0/10	0/10	5/9
Spinal meninges	0/3	3/10	3/10	7/12	0/3	2/10	4/10	4/11
Peripheral nerves	0/3	0/3	3/3	3/3	0/3	0/3	2/3	1/3
Encephalitis^b^	0/3	10/10	10/10	15/15	0/3	1/10	5/10	10/11
Gliosis in brain^c^	0/3	10/10	0/10	4/15	0/3	0/10	3/10	0/11

^a^Indicates anatomical areas affected by inflammation, with the exception of “gliosis” category.

^b^Encephalitis, as tallied in birds with inflammatory lesions in any brain section (cerebrum, optic lobe, brainstem, cerebellum).

^c^Gliosis, as tallied in birds with proliferation/hypertrophy of glial cells or glial nodules in any brain section (cerebrum, optic lobe, brainstem, cerebellum).

^d^Number of birds with disease in that anatomical location/number of birds tested at that time point.

In the IC group, inflammation of the central nervous system (CNS) was not present at 1 week post infection (wpi), however by 4 wpi, 100% and 70% of birds presented with encephalitis and myelitis, respectively; a frequency that remained approximately constant through the end of the experiment. In this group, inflammation of the peripheral nervous system (PNS; axillary and ischiatic nerves) was detected at 8 and 12 wpi in 100% of birds. The cerebrum, optic lobe, and brainstem had the highest inflammation scores at 4 wpi. The inflammation in the cerebrum at 4 wpi was significantly more severe compared to the cerebrum, spinal cord, and cerebellum at all the other time points. Inflammation in the optic lobe at 4 wpi was significantly more severe compared to the cerebrum at 8 wpi, as well spinal cord and/or cerebellum at all other time points. Encephalitis in the brainstem was more severe compared to inflammation of the spinal cord at 4 and 8 wpi, as well as the cerebellum at 12 wpi. No significant score differences were seen between areas of the central nervous system within the same time point at 8 and 12 wpi (Fig. 1a).

In the IM group, inflammatory lesions were not present at 1 wpi, and at 4 wpi encephalitis and myelitis were present only in 10% and 30% of birds. By 12 wpi frequency of inflammation at these sites had increased to 91% and 64%, respectively. Peripheral neuritis was observed at 8 and 12 wpi, and it never affected more than 67% of the birds. In the IM group, low-intensity inflammation was initially recorded only in the cerebrum and spinal cord at 4 wpi. Lesions became more severe at 8 and 12 wpi, with inflammation in the cerebrum at 12 wpi being significantly more severe than at 4 wpi (Fig. 1b).

When the brain scores (calculated by averaging the inflammation of each available brain area) were considered, birds in the IC group at 4 wpi presented significantly more severe lesions compared to birds in the IM and IC groups at all time points (Fig. 2). In the IM group, inflammation gradually became more severe as time progressed, with differences being significant between 4 and 12 wpi. These results show that Muscovy ducks inoculated intracranially with ABBV-1 develop more severe lesions at earlier time points compared to birds inoculated through the IM route, and that the peak of inflammation in birds from the IC group occurs at 4 wpi, while a peak of inflammation could not be identified in the IM group.

#### Morphology of microscopic lesions

**Figure 3:**
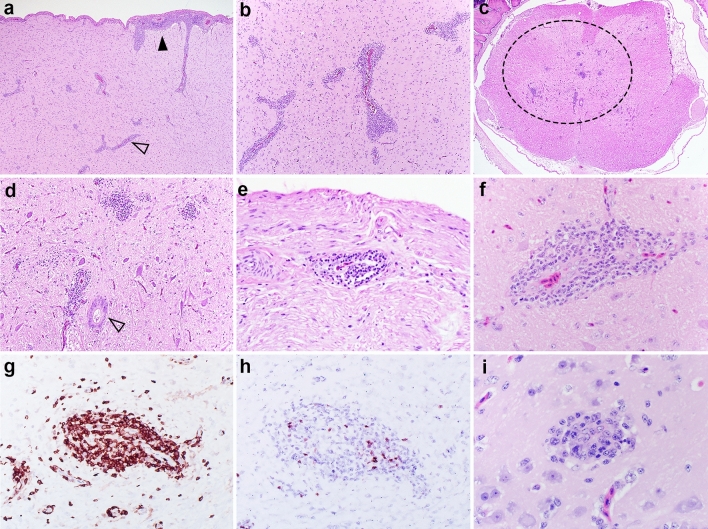
Histology of representative lesions in the nervous tissue of Muscovy ducks experimentally infected with aquatic bird bornavirus 1 (ABBV-1). (**a**) Cerebrum, duck #519 IC group, sampled at 4 weeks post infection (wpi). The leptomeninges (black arrowhead) and Virchow-Robin spaces (empty arrowhead) are segmentally and multifocally expanded by an inflammatory infiltrate. HE, original magnification 100X. (**b**) Cerebrum, duck #519 IC group, 4 wpi. The perivascular spaces are expanded by a severe mononuclear inflammatory infiltrate. HE, original magnification 40X. (**c**, **d**) Spinal cord, duck #612 IM group, 8 wpi. Mainly affecting the grey matter (circle), there are multifocal areas of inflammation composed of mononuclear cells. Empty arrowhead indicates the central canal. HE, original magnification 40X (**c**) and 100X (**d**). (**e**) Peripheral nerve (ischiatic or axillary), duck #621 IM group, 8 wpi. There are scattered inflammatory perivascular cuffs in the endoneurium of affected nerves. HE, original magnification 200X. (**f**) Cerebrum, duck #502 IC group, 12 wpi. The perivascular cuffs are composed of mononuclear cells, predominately lymphocytes with scattered plasma cells. HE, original magnification 400X. (**g**, **h**). Optic lobe, duck #502 Immunohistochemistry for CD3 (**g**) and Pax-5 (**h**) antigens on consecutive sections. The large majority of inflammatory cells displays strong cytoplasmic reactivity for CD3 antigen (T lymphocytes); while scattered cells display intranuclear reactivity for Pax-5 (B lymphocytes). Reactivity for CD3 clearly shows spilling of T lymphocytes from the perivascular cuffs into the adjacent neuroparenchyma. IHC, DAB chromogen with hematoxylin counterstain, original magnification 200X. (**i**) Cerebrum, duck #534 IC group, 4 wpi. There are rare, randomly distributed, accumulations of glial cells arranged in small clusters (glial nodules). These cells are round with moderate amounts of eosinophilic cytoplasm and open chromatin. HE, original magnification 400X.

Inflammation did not appear to target specific areas of the nervous tissue, and was characterized by accumulation of a mononuclear infiltrate that expanded the perivascular spaces in the brain and spinal cord, segmentally the meninges, and the endoneurium of the axillary or ischiatic nerves (Fig. 3a–e). Within the brain, both the grey and white matter were affected and in the spinal cord predominantly the grey matter (Fig. 3c,d). Inflammatory cells were predominately lymphocytes, with fewer macrophages, and rare plasma cells and heterophils (Fig. 3f). Immunohistochemistry (IHC) conducted on representative duck brains (n = 3) from the IC group at 12 wpi showed that the highest percentage of the inflammatory population was composed of CD3-positive cells (T lymphocytes), with a small percentage of Pax-5-positive cells (B lymphocytes) (Fig. 3g,h).

Besides inflammation, gliosis characterized by proliferation and hypertrophy of glial cells with formation of glial nodules (Fig. 3i) was seen in 100% and 27% of IC birds at 4 and 12 wpi, respectively, and in 33% of IM birds at 8 wpi (Table 1). Inflammation was not identified in the myenteric nerves or ganglia of the gastrointestinal tract. No significant microscopic findings were identified in the other tissues from the IC and IM groups, and no lesions were identified in the birds from the PO and CO groups.

### Detection of ABBV-1 RNA in tissues

**Figure 4:**
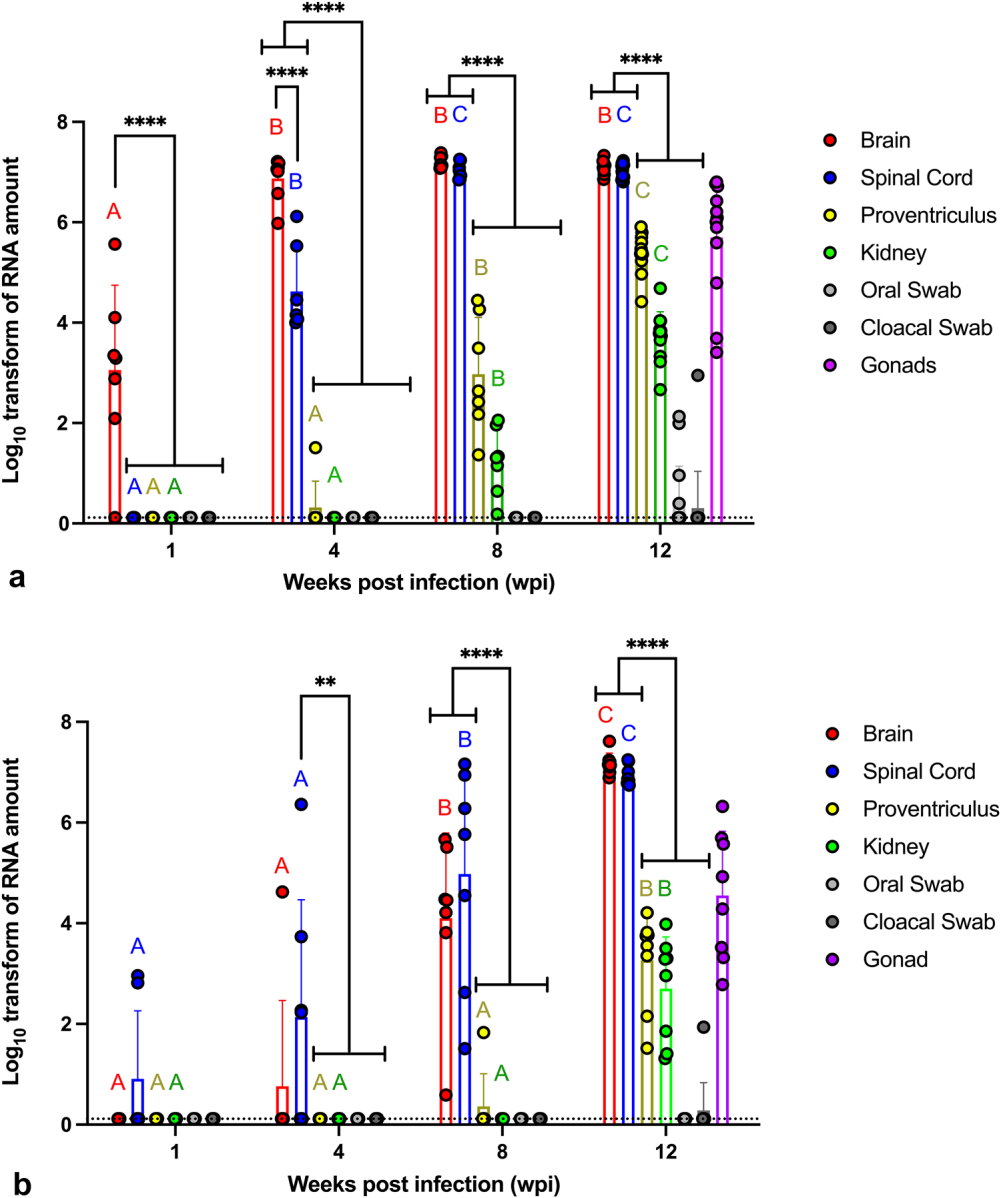
Scatter plot of virus RNA copies in multiple tissues and swabs from Muscovy ducks experimentally infected with ABBV-1 through the intracranial (**a**) or intramuscular (**b**) routes of infection, and assessed at 1, 4, 8, and 12 weeks post infection (wpi). Negative data points are represented at the calculated limit of detection (dotted line). Significant differences between terms *within* the same time point are identified with simple binary connectors. Flat lines at the end of a connector indicate differences with multiple underlying terms (significance level is reported for the highest p value among the comparisons). Differences for the same organ *between* time points are identified by different letters and color-coded (cerebrum, red; spinal cord, blue; proventriculus, yellow; kidney, green). Single datapoints represent the log_10_-transform of the virus RNA copies/150 µg of total RNA (tissues), or 84 µl of swab liquid. Columns represent mean + standard deviation. Two-way ANOVA with Tukey’s test for multiple comparisons (* < 0.05, ** < 0.01, *** < 0.001, **** < 0.0001). The limit of detection was calculated by extrapolating the value of the virus RNA copies corresponding to a Ct value of 35, based on the standard curve. Data points for gonads, as available only at 12 wpi, were not included in the statistical analysis.

Virus RNA copies were quantified by reverse transcriptase quantitative polymerase chain reaction (RT-qPCR), in order to test the magnitude of virus replication in brain, spinal cord, proventriculus, kidneys, and gonads, and shedding through the choanal and cloacal secretions (Table 2 and Fig. 4). In both the CO and PO groups, none of the tissues or swabs tested positive for ABBV-1 RNA at any time points. In the IC group, at 1 wpi the brain was the only tissue to test positive in 6 of 7 birds. By 4 wpi, and until the end of the experiment, both the brain and the spinal cord were positive in 100% of tested birds. At 4 wpi, one bird had the proventriculus positive (1/7), and by 8 wpi to the end of the experiment all proventricular tissues tested positive. At both 8 and 12 wpi, 100% of renal tissues were positive, and at 12 wpi all gonads were also positive (4 testes and 8 ovaries). There was evidence of shedding of viral RNA in 27% and 7% of choanal and cloacal swabs at 12 wpi (Table 2). The average concentration of virus RNA copies in the brain of IC birds significantly increased from 1 wpi (10^3^/150 ng of total tissue RNA) to 4 and 8 wpi (10^7^ and 10^7.2^), after which remained approximately constant (12 wpi). Similarly, virus RNA copies in the spinal cord significantly increased approximately 2 orders of magnitude from 4 wpi (10^4.6^) to 8 wpi (10^7^), and remained constant at 12 wpi. Overall, the tissues from brain and/or spinal cord contained significantly more viral genetic material compared to all the other samples at 4, 8, and 12 wpi (Fig. 4a).

**Table 2:** Number of birds in the intracranial (IC) and intramuscular (IM) group, which tested positive for the ABBV-1N gene in a cohort of select tissues at 1, 4, 8, and 12 weeks post infection (wpi).

Samples	IC				IM			
	1 wpi	4 wpi	8 wpi	12 wpi	1 wpi	4 wpi	8 wpi	12 wpi
Brain	6/7^a^	7/7	7/7	12/12	0/7	1/7	7/7	8/8
Spinal cord	0/7	7/7	7/7	12/12	2/7	4/7	7/7	8/8
PV^b^	0/7	1/7	7/7	12/12	0/7	0/7	1/7	8/8
Kidney	0/7	0/7	7/7	12/12	0/7	0/7	0/7	8/8
Gonad	NA^c^	NA	NA	12/12	NA	NA	NA	8/8
Cloacal swab	0/10	0/10	0/10	1/15	0/10	0/10	0/10	1/11
Choanal swab	0/10	0/10	0/10	4/15	0/10	0/10	0/10	0/11

^a^Indicates number of birds positive for that tissue/total number of birds tested for that tissue at that time point.

^b^PV, proventriculus.

^c^NA, the tissue was not available for analysis.

In the IM group, at 1 wpi the spinal cord was the only tissue to test positive in 2 of 7 birds. At 4 wpi, the spinal cords of 57% of birds tested positive, and one bird tested positive also in the brain. By 8 wpi all birds tested positive in the spinal cord and brain, and one bird was positive in the proventriculus. At 12 wpi, the proventriculus, kidneys, and gonads (3 testes and 5 ovaries) from all birds were positive, and there was evidence of viral shedding in one cloacal swab only (1/11) (Table 2). The average concentration of virus RNA copies in the spinal cord gradually increased from 1 to12 wpi, when it averaged approximately 10^7^ virus RNA copies/150 ng of tissue RNA, and was significantly higher compared to all the other time points. In the brain, virus RNA copies were first detected at 4 wpi, and gradually increased up to 12 wpi, when the amount was significantly higher compared to all the other time points. The tissues from the brain and/or spinal cord contained significantly more viral genetic material compared to all the other samples at 4, 8 and 12 wpi (Fig. 4b).

### Relationship between virus RNA in tissues and brain pathology

A multivariable linear regression analysis was used to predict brain and spinal cord inflammation scores in both the IC and IM groups, using as explanatory variables the amounts of virus genetic material in organs (brain, spinal cord, proventriculus) and time post infection (wpi). Univariable (unconditional) association analyses showed that only the brain inflammation score in the IC group was significantly and negatively correlated to all 4 predictors, as also indicated by Spearman’s correlation analysis (Supplementary Figure S2). The other variables (i.e., spinal cord inflammation score in the IM and IC groups, and brain inflammation score in the IM group) either had no significant correlations with the predictors or did not meet the linearity assumption. Therefore, a multivariable regression model was built for the brain pathology score in the IC group only, which showed that the 4 predictors explained 64.9% of inflammation variability (R^2^ = 0.649, F(4,21) = 9.72, *P* <  0.001). Only the quantity of virus RNA in the brain significantly predicted pathology scores of the brain (ß coefficient = - 1.522; *P* =  0.001), indicating that for every tenfold increase of virus RNA copies in the brain, the predicted pathology brain score decreased by 1.522 (Table 3).

**Table 3:** Multivariable linear regression analysis using the brain pathology score as the dependent variable, and virus RNA copies in selected tissues and week post infection as independent variables, as assessed in a cohort of Muscovy ducks infected with aquatic bird bornavirus (ABBV)-1 delivered intracranially.

Predictor	Coefficient	Standard error	*P* value	95% confidence intervals	
*Week post infection*^a^	0.129	0.219	0.561	− 0.326	0.584
*Brain titer*^b^	− 1.522	0.406	0.001	− 2.367	− 0.677
*Spinal cord titer*	− 0.107	0.159	0.506	− 0.438	0.223
*Proventriculus titer*	− 0.107	0.064	0.111	− 0.241	0.027

^a^Only 4, 8 and 12 week post infection were evaluated in the analysis.

^b^Titer in tissues is expressed as log10 of virus RNA copies per 150 ng of tissue RNA.

### Immunohistochemistry

Seven birds from the IC group at 12 wpi, plus one control from the same time point, were submitted to detect the distribution of ABBV-1N protein in tissues by IHC. Birds from the IC group at 12 wpi were chosen, as this group had the highest ratio of ABBV-positive organs and virus RNA copies, as assessed by RT-qPCR. All infected birds had characteristic histologic lesions in the brain and spinal cord, while controls presented no lesions. The N antigen was detected in all seven infected birds (Table 4).

**Table 4:** Distribution of tissue reactivity for aquatic bird bornavirus N protein in seven ducks infected with ABBV-1 through the intracranial route 12 weeks post infection (wpi).

Tissues	#1	#2	#3	#4	#5	#6	#7	Total	Cells positive for ABBV antigen^e^
Cerebrum	3^a^	1	3	2	2	3	3	7/7^b^	Neurons, glial cells, Purkinje cells, ependymal cells
Optic lobe	2	2	1	NA^f^	NA	NA	NA	3/3	
Brainstem	1	1	2	NA	NA	NA	NA	3/3	
Cerebellum	2	2	3	2	NA	2	3	6/6	
Spinal cord	1	2	1	NA	3	2	2	6/6	Neurons, glial cells, ependymal cells
Peripheral nerves	+^c^	+	−	NA	NA	NA	NA	2/3	Ganglia, nerve fibers, glial cells
Kidney	NA	−	−	−	−	−	−	0/6	−
Gonad	+ (M)^d^	NA	+ (M)	+ (F)	+ (M)	+ (F)	+ (F)	6/6	Interstitium of testes and ovaries, theca, and granulosa cells
Proventriculus	+	+	+	+	+	+	+	7/7	Glands, myenteric plexuses
Ventriculus	+	+	NA	NA	+	+	+	5/5	Myenteric plexuses
Heart	−	−	−	NA	NA	NA	NA	0/3	−
Pancreas	+	−	+	NA	NA	NA	NA	2/3	Acinar cells, ganglia
Adrenal	+	NA	+	NA	NA	NA	NA	2/2	Cortical cells, medullary cells
Thyroid/parathyroid	−	NA	−	NA	NA	NA	NA	0/2	−
Small intestine	+	+	+	NA	NA	NA	NA	3/3	Submucosal and myenteric plexuses
Colon	+	+	+	NA	NA	NA	NA	3/3	Submucosal and myenteric plexuses
Lung	+	−	+	NA	NA	NA	NA	2/3	Ganglia
Liver	−	−	−	NA	NA	NA	NA	0/3	−
Spleen	−	−	−	NA	NA	NA	NA	0/3	−
Trachea	−	−	−	NA	NA	NA	NA	0/3	−
Esophagus	NA	+	−	NA	NA	NA	NA	1/2	Submucosal and myenteric plexuses
Bursa	−	NA	−	NA	NA	NA	NA	0/2	−
Thymus	−	NA	−	NA	NA	NA	NA	0/2	−

^a^Indicates IHC distribution score for cerebrum, optic lobe, brainstem, cerebellum, and spinal cord.

^b^Indicates the total number of birds with immunohistochemical reactivity in that tissue/total number of birds with available tissue for testing.

^c^Indicates presence (+) or absence (−) of immunohistochemical reactivity in the tissue (without distribution score).

^d^In parenthesis, male (M) or female (F) sex of the bird is indicated.

^e^Description of the type of positive cells.

^f^NA = tissue was not available for analysis.

**Figure 5:**
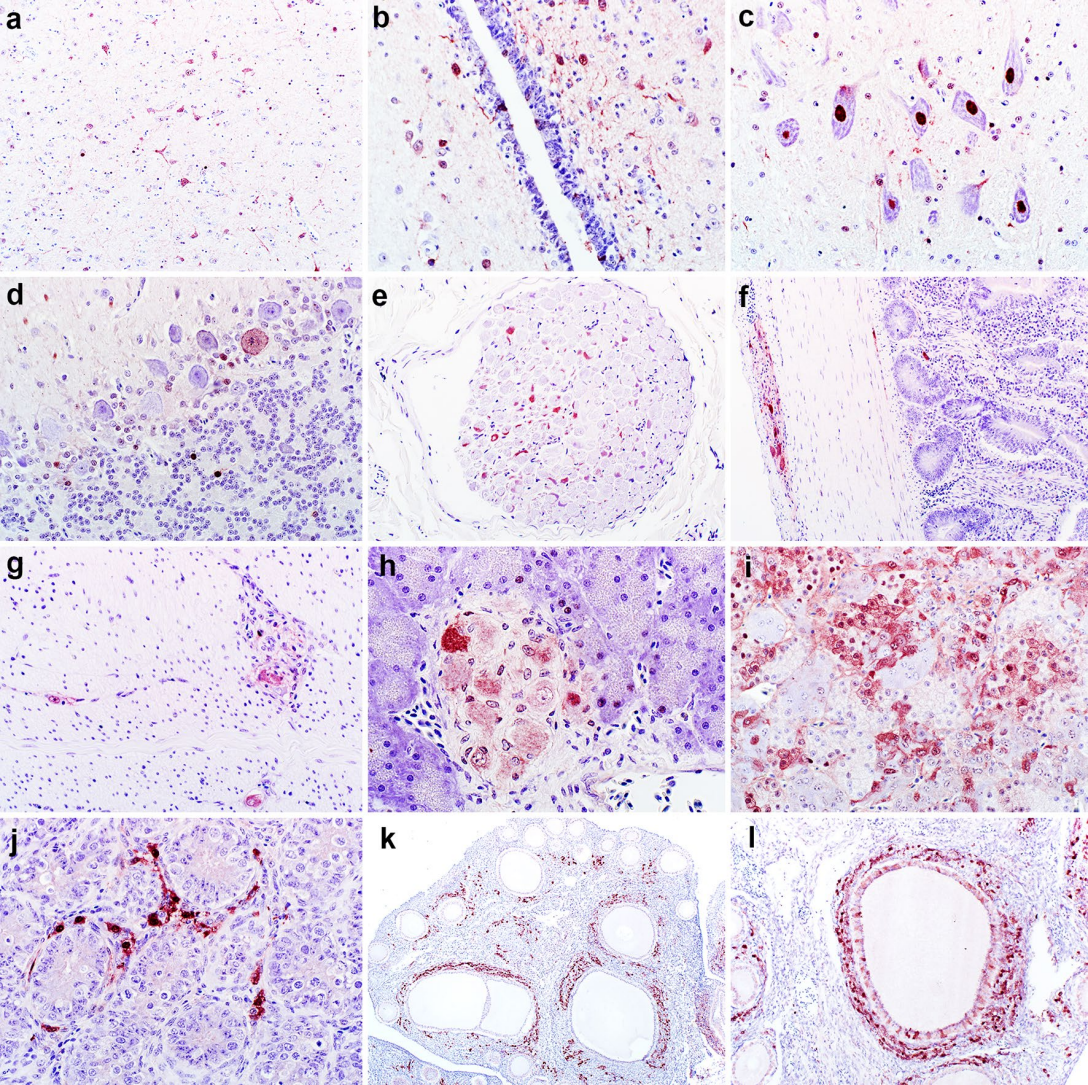
Representative immunohistochemical (IHC) reactivity for ABBV-1 nucleoprotein (N) in tissues of Muscovy ducks experimentally infected intracranially with ABBV-1 and sampled at 12 weeks post infection (wpi). (**a–d**) Immunoreactivity for ABBV-1N is observed in scattered neurons and glial cells in the cortex of the forebrain (**a**, duck #502), periventricular neuroparenchyma and rare ependymal cells (**b**, duck #537), in clusters of large neurons in the medulla oblongata (**c**, duck #543), and in scattered Purkinje and granular cell in the cerebellum (**d**, duck #502). Reactivity is most often intensely nuclear, with rare light cytoplasmic signal. (**e–l**) In the peripheral nervous tissue, immunoreactivity is observed in the axons of peripheral nerves (**e**, duck #502), myenteric plexuses of the small intestine (**f**, duck #542), and ventriculus (**g**, duck #502), ganglia and acinar cells of the pancreas (**h**, duck #502), and medullary and cortical cells of the adrenal gland (**i**, duck #502). (**j–l**) In the testis, reactivity is observed in the interstitial cells between tubules (**j**, duck #502), and in the ovary, reactivity is present in the interstitial cells between follicles, theca cells, and granulosa cells (**k–l**, duck #542).

In the CNS, immunolabeling in the neuroparenchyma was multifocal, and not spatially associated with regions with inflammation, as observed histologically (Fig. 5a). Reactivity of moderate intensity was present in the nuclei and/or nuclei and cytoplasm of predominantly neurons and glial cells, while ependymal cells were also occasionally positive (Fig. 5b–d). A scoring based on the extent of IHC immunolabeling showed no significant differences between sections of the brain (Kruskal–Wallis test; data not shown).

Outside of the central nervous system, immunoreactivity was found in peripheral nerves (Fig. 5e), ganglia and plexuses of the proventriculus, ventriculus, esophagus, small intestine, colon (Fig. 5f,g), lung, and pancreas (Fig. 5h). In addition, scattered epithelial cells of the proventricular glands, acinar cells of the pancreas, and cortical and medullary cells of the adrenal gland (Fig. 5i) exhibited positive intranuclear and occasionally intracytoplasmic immunoreactivity. In all three tested males, reactivity was seen in the interstitial cells (Fig. 5j) and the epithelium of the epididymis. In the three tested females, reactivity was identified in scattered cells of the theca interna and externa, rare granulosa cells, and the ovarian interstitium (Fig. 5k,l), besides neurons in adjacent ganglia. Immunoreactivity was not observed in kidney (despite high RT-qPCR signal), heart, liver, spleen, trachea, bursa of Fabricius, or thymus in any of the examined birds. The control duck exhibited no immunolabeling in any of the examined tissues.

### Serology

**Figure 6:**
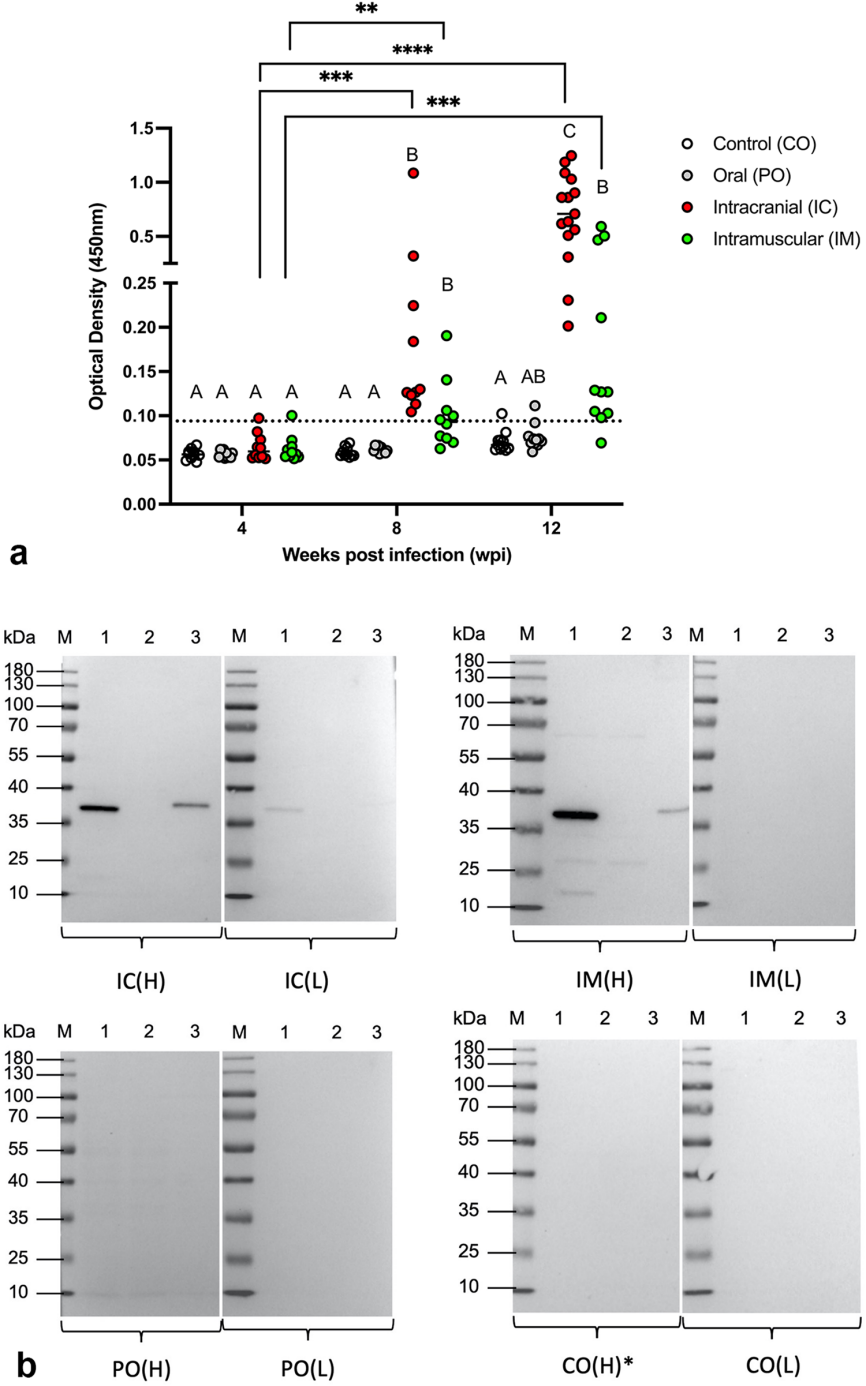
(**a**) Magnitude of seroconversion in Muscovy ducks experimentally infected with aquatic bird bornavirus-1 (ABBV-1) through the intracranial (IC), intramuscular (IM) and oral (PO) inoculation routes, at 4, 8 and 12 weeks post infection (wpi). Control birds (CO) received carrier only. Seroconversion was determined by ELISA against the ABBV-1N protein. Data are represented as the scatterplot of the optical densities (ODs) normalized to a plate calibrator (see methods), and each value is the average of technical triplicates. The cut-off value (dotted line) to determine positive birds was calculated as the average + three-times the standard deviation of the values from all CO groups. Significant differences between time points are identified by connectors; differences within the same time point are identified by different letters. Krurskal-Wallis test with Benjamini, Krieger, and Yekutieli procedure for false discovery rate (* < 0.05, ** < 0.01, *** < 0.001, **** < 0.0001). (**b**) Representative immunoblots to identify presence of immunoglobulins against ABBV-1 nucleoprotein (N) in the serum of Muscovy ducks from the IC, IM, PO and CO groups at 12 wpi. For each experimental group, one serum with highest (H) and one with lowest (L) OD values (as assessed by ELISA) was used. Depicted are blots carried out using the sera from IC ducks (top left), IM (top right), PO (bottom left), and CO (bottom right). For each blot, lane 1 was loaded with the recombinant N protein used to coat the ELISA plates; lane 2 was loaded using a cell lysate from non-infected CCL-141 cells; lane 3 was loaded using a cell lysate from CCL-141 cells persistently infected with ABBV-1. (M) ladder. The ABBV-1N protein migrates between 40 and 30 kDa; birds with serum identifying a band of this molecular size are considered seropositive. *Indicates serum from the CO birds (#821) with an OD above the threshold. Pictures of the original membranes are available in the Supplementary Material.

Sera from all groups at 4, 8, and 12 wpi were tested by ELISA for antibodies against the ABBV-1N protein. The raw optical density (OD) values were normalized to calibrators across all plates. After normalization, a cut-off OD value was determined for all time points, and calculated as the average OD of the control (CO) birds plus three-times the standard deviation (SD). According to this threshold, at 4 wpi, only one bird had seroconverted in the IC and IM groups. At 8 wpi, 10/10 and 5/10 birds had seroconverted in the IC and IM groups, respectively. At 12 wpi, 15/15, 10/11, and 1/11 birds had seroconverted in the IC, IM and PO groups, respectively. At each time point, the highest magnitude of seroconversion was observed in the IC group, although this was not significantly higher compared to the IM group, likely due to high data dispersion. Overall, the number of birds that seroconverted and the magnitude of seroconversion increased from 4 to 12 wpi in both the IC and IM groups (Fig. 6a). Notably, one CO bird at 12 wpi had an OD marginally above (0.1) the calculated threshold (OD, 0.094) (Fig. 6a). This was interpreted as an outlier, and possibly caused by increased non-specific background. Older CO birds, however, appeared to have a slight increase of OD values; the reason for this is unclear and possibly linked to the quality of the collected sera.

Selected sera in the IC and IM groups, which had the highest and lowest OD values at 12 wpi by ELISA, were used in immunoblots to detected the purified recombinant ABBV-1 N protein or the N protein from cell lysates of ABBV-1-infected CCL-14 cells (Fig. 6b). From the IC group, the high OD serum highlighted two intense band, while the low OD serum showed a very faint band only in the purified protein lane. From the IM group, the high OD serum showed two bands, while none were observed using the serum from the only bird with an OD value below the threshold. No sera from the PO and CO birds detected ABBV-1N protein in immunoblots (Fig. 6b). Notably, CO duck #821 (the outlier in the ELISA) did not detect any band, supporting the notion that the OD increase in the CO group is likely non-specific. Lastly, immunoblots showed that the antigen used for coating the ELISA plates was recognized by the monospecific antibody against ABBV-1N protein used in IFA and IHC (Supplementary Figure S3).

### Virus isolation from brains and kidneys

Viable brains from all birds in the IC and IM groups at 12 wpi were used for virus isolation. In the IC and IM groups, respectively, 8/11 and 8/8 brains yielded ABBV-1 in CCl-141 cells after up to four passages, as shown by nuclear and weak cytoplasmic signal in scattered cells by IFA. Although 12 birds were in the IC group, one brain could not be tested due to poor sample preservation. Similarly, the kidneys from 2 ducks in the IC group at 12 wpi, which tested positive for RT-qPCR but were negative by IHC, yielded ABBV-1 in CCl-141 cells after three passages (Supplementary Figure S4). The control brains (n = 4) and kidney (n = 1) from the CO group at 12 wpi yielded no virus.

## Discussion

This study describes for the first time the successful experimental infection of an avian species with ABBV-1, and confirms the ability of this virus to infect commercial waterfowl. Intracranial and intramuscular infection of Muscovy ducklings with ABBV-1 led to viral spread and replication in multiple tissues and development of nervous lesions analogous to those observed in natural cases.

To determine viral replication upon infection, ABBV-1 RNA copies was evaluated in multiple tissues at 1, 4, 8, and 12 wpi. ABBV-1 RNA was detected in all tissues from birds in the IC and IM groups by 12 wpi. In the IC infected ducks, viral RNA was identified in the brain as soon as 1 wpi. By 4 wpi until the end of the experiment all tested brains were positive. These findings indicate intracranial inoculation is a very effective method of ABBV-1 infection and is in agreement with other studies, which documented the intracranial route as a successful delivery method to infect rodents and psittacine birds with BoDV-1 and PaBVs^27,28^, respectively. By 4 wpi, ducks in the IC group showed viral replication in the spinal cord, and by 12 wpi all tested peripheral organs (kidney, proventriculus, gonads) were also positive. This time-wise spread of the virus from the brain to the spinal cord and to the visceral organs is suggestive of a centrifugal spread from the central nervous tissue to the periphery. Conversely, in the IM group, ABBV-1 RNA was first identified in the spinal cord at 1 wpi. By 8 wpi, all tested spinal cords and brains were positive, and by 12 wpi all peripheral tissues were positive. This kinetic suggests a centripetal spread from the muscle to the spinal cord and brain, as well as a centrifugal spread from the central nervous tissue to visceral organs, and is consistent with reports describing experimental intramuscular inoculation of PaBVs in psittacine birds^24,32^. Lastly, infectious virus was successfully re-isolated from 73 and 100% of the brains of birds in the IC and IM group, respectively, indicating that RNA signal corresponded to infectious virus. It is unclear why virus isolation was unsuccessful in three birds from the IC group, despite presence of virus RNA and IHC reactivity on section. It is possible that poor tissue preservation (e.g., incomplete penetration of RNA preservation solution) in these samples might have played a role.

Ducks inoculated orally could not become infected, as shown by lack of ABBV-1 RNA in any collected tissues. Oral administration of PaBVs^23^ and BoDV-1^33^ in cockatiels and rats, respectively, did not yield infection or clinical signs, in agreement with our findings. Difficulty in reproducing successful infection through oral administration has led to questioning fecal–oral transmission, which had been postulated as the natural route of infection for bornaviruses in birds^34^. In a recent study, cockatiels were infected with PaBV by applying the infectious inoculum through defects in the skin of the footpad, suggesting that wound contamination may be a natural way for PaBV infection to occur^35^; in this case infection would depend on the levels of environmental contamination, rather than bird-to-bird transmission. In our study, only a few birds (n = 6) tested positive for ABBV-1 RNA in the choanal and/or cloacal swabs at 12 wpi, indicating the potential for virus shedding and environmental contamination. Moreover, all tested kidneys from ducks in both the IC and IM groups at 12 wpi were positive for ABBV-1 RNA, suggesting that shedding through the urine is plausible. This is consistent with successful demonstration of PaBV RNA in the urine of experimentally and naturally infected psittacine birds^36^. Despite establishment of persistent infection in all ducks in the IC and IM groups (including renal tissues), however, only a few swabs tested positive with low amounts of virus RNA, consistent with intermittent and low-intensity viral shedding. This is partially different from what is observed in psittacine birds, which can shed high amounts of virus RNA for long periods^37,38^. While presence of ABBV-1 RNA in the swabs indicates potential for horizontal transmission, presence of genetic material does not necessarily correlate with presence of infectious viral particles, and even if shedding of infectious virus particle were to be conclusively demonstrated, it still remains to be explained why the oral route is ineffective in establishing experimental infection.

Vertical transmission is another potential route of ABBV-1 infection. In our Muscovy ducks, immunoreactive ABBV antigen was present in the interstitial cells of the testis, epithelium of the epididymis, ovarian granulosa cells, and interstitial stromal cells. Presence of virus protein in cells that can be in contact with the sperm or ovum suggests that vertical transmission may be possible. Similarly, PaBV and CnBV-2 antigen has been demonstrated in testes, ovaries, and shell gland of psittacine and canary birds, both naturally and experimentally infected^26,31,39^. In conures, PaBV-2 was even detected in the embryos from persistently infected breeders and in the blood of one hatchling^40^, and another study reported the presence of ABBV-1 RNA in the egg yolk of one out of 53 non embryonated Canada goose eggs^41^. While in our study female ducks did not reach sexual maturity and eggs could not be tested for ABBV-1, it should be noted that even if the virus can reach the egg, actual infection of the embryo may not necessarily follow. In our experience, experimental inoculation of fertile Pekin duck eggs with purified ABBV-1 through the yolk sac and allantoic fluid was not able to successfully infect the embryos^42^.

Viral distribution was also evaluated by IHC, with the goal to integrate the results of RT-qPCR and document the widest possible extent of infected tissues and cell types. Tissues from IC birds at 12 wpi were chosen, as this group presented with the broadest virus distribution and highest magnitude of virus RNA in tissues, as shown by RT-qPCR. Consistent intranuclear—with or without cytoplasmic immunoreactivity—was present in neurons, glial cells, and ependymal cells of the central nervous system. Immunolabeling for ABBV was also identified in numerous visceral organs, in agreement with detection of ABBV-1 RNA in proventriculus, testes, and ovaries by RT-qPCR. Virus distribution outside of the CNS and in visceral organs is consistent with natural reports of ABBV infections^9^, and multiple accounts of experimental PaBV infection in psittacine birds^39,43,44^. None of the visceral tissue with IHC signal showed inflammatory lesions, as well, inflammation in the CNS did not overlap with areas of IHC reactivity. In an experimental infection study of parrot bornavirus 2 (PaBV-2) in cockatiels, immunolabeling for PaBV N-protein preceded the development of inflammation throughout all time points^32^. It is possible that a longer duration of our study may have been needed for inflammatory lesions to overlap with areas of ABBV replication. Notably, while kidneys had large amounts of ABBV-1 RNA copies and virus was re-isolated from the two tested kidneys, no IHC reactivity was observed in the sections from renal tissue. Reports of natural and experimental PaBV infection indicate that bornavirus immunoreactivity is often observed in the renal tubular epithelium^26,39^. The reason for this discrepancy is unclear; and may be caused by low-level expression of virus protein in renal tissues of the ducks in our study^30,45^, which may be below the sensitivity threshold of IHC. Similarly, no epithelial cells in the gastrointestinal tract (with the exception of rare proventricular glands) showed immunoreactivity for ABBV-1, suggesting that virus replication is limited to the mural ganglia, and that virus shedding through loss of infected epithelial cells in the intestinal lumen is not a common route of virus dissemination. Limited virus replication in the intestinal and renal epithelia may explain the low-level amounts of virus RNA detected in the cloacal swabs of our infection model.

In this study, the experimentally infected ducks in the IC and IM group developed histologic lesions exclusively in the nervous system, such as lymphocytic encephalitis, myelitis, meningitis, and peripheral neuritis, which are consistent with descriptions of natural ABBV-1 infection in waterfowl^9^. Other features described in natural infection^9^, including Wallerian degeneration, malacia, cerebral edema, and inflammation of the autonomic nervous system, were not appreciated. While ganglioneuritis is the characteristic histological lesion in cases of proventricular dilation disease, it appears as a less common finding in cases of ABBV-1 infection, although still described^6,9^.

In the IC group, the overall brain inflammation score peaked at 4 wpi and decreased at later time points, despite the concentration of virus RNA in the brain remaining constant or even slightly increasing. This is confirmed by regression analysis, which showed that increasing virus RNA copies in the brain corresponded to a decrease in brain pathology scores. In the IM ducks, the overall brain inflammation score gradually increased over time, as virus RNA concentration increased, but an inflammatory peak could not be observed for the duration of the study. In the spinal cord, inflammation was first observed at 4wpi, and the peak of inflammation was seen at 8 wpi, while the highest virus RNA amount in this organ was detected at 12 wpi. Therefore, encephalitis and myelitis follow the pattern of initial ABBV-1 RNA detection, but the magnitude of inflammation does not appear to be related to the amount of virus RNA in tissues.

Despite development of histologic lesions characteristic of ABBV-1 infection and demonstrating viral replication in multiple tissues, ABBV-1 experimentally infected Muscovy ducks did not show macroscopic lesions (e.g. proventricular dilation) or develop clinical signs at any point during the 12-week duration of this study. While clinical signs attributed to natural ABBV-1 infection in wild waterfowl include poor body condition, gastrointestinal signs, and neurologic deficits^9^, ABBV-1 has been identified in tissues of apparently healthy waterfowl^17,18^. In two different studies of canaries experimentally infected with CnBV-1 and 2, regardless of route of administration (IM, PO, subcutaneous, nasal), birds did not develop any clinical signs for the 154-day and 161-day duration of the studies, respectively, despite having high loads of virus RNA in multiple tissues (e.g. brain, proventriculus, lung, heart, liver, duodenum, and kidney) as assessed by RT-qPCR^29,31^. Experimental infection of PaBV-4 intramuscularly in adult cockatiels did not yield clinical signs until 92 dpi^26^. It is unclear what triggers may promote development of clinical disease in persistently infected birds. The pathogenesis of the inflammation in psittacine birds infected with PaBV has been proposed to be associated with a T cell-mediated immune mechanism, rather than by direct virus damage to the infected cells^46,47^. This is supported, for instance, by the fact that birds treated with cyclosporin become infected but do not develop clinical signs^46^. A recent article has shown that hatchling cockatiels infected with PaBV-4 became readily infected and developed encephalitis but no clinical signs, as opposed to older birds which developed both severe encephalitis and clinical signs^22^. These findings are very similar to what is reported in our study, and suggest that infection of ducklings with an immune system that is not fully developed^48^ may promote immune-tolerance and a carrier state^22^. In our cohort, 100% and 91% of ducks in the IC and IM groups, respectively, had seroconverted by 12 wpi, suggesting that birds in our cohort were sufficiently immune-competent to develop humoral immunity; however, the magnitude of antibody response is not a proxy to gauge the degree of activation of the cell-mediated immunity. Despite antibody production at 12 wpi, especially in the IC group, virus infection was not cleared. This is consistent with the fact that the N protein is not a neutralizing antigen, and agrees with antibody kinetics of persistent bornavirus infection in other avian species^37,38^.

The Muscovy ducks used in this study were obtained from a local hatchery in Southern Ontario. As ABBV is known to be widespread in free-ranging waterfowl in the area^9,15^, we considered the possibility that these commercial ducklings could already have been infected when obtained at 1-day old. However, all birds in both the CO and PO group, at no point throughout the duration of the study demonstrated viral RNA in any tested tissues or swabs, nor did they develop histologic lesions in contrast to the IC and IM inoculated birds, making us confident these ducks were not infected prior to inoculation. Additionally, as birds were received in the isolation facilities at day of age from a hatchery, the potential for infection with ABBV-1 would have been minimal.

## Conclusions

Muscovy ducklings could become infected with ABBV-1 by intracranial and intramuscular, but not oral, inoculation routes. Despite virus replication in multiple tissues, development of inflammatory lesions in the central nervous system, and seroconversion, infected birds did not develop clinical signs. Shedding of virus was low and likely intermittent, suggesting that low-rate environmental contamination by infected birds may be possible. This is the first documented experimental infection with ABBV-1, and indicates that the Muscovy duck can reproduce some of the features of natural ABBV-1 infection. However, lack of clinical signs and minimal virus shedding suggest that additional experimental variables (e.g., age at infection and duration of experimental trial) could be modified to improve the relevance of this model and more closely reproduce ABBV-1 infection as observed in the field. Lastly, as the outcome of bornavirus infection is highly variable based on the host, one should consider the possibility that experimental infection of Muscovy ducks may not be representative of infection in other waterfowl or poultry species.

## Materials and methods

### Cells

Immortalized duck embryo fibroblasts (CCL-141), originated from Pekin duck (*Anas platyrhynchos domesticus*), were purchased from the American Type Culture Collection (ATCC) and passaged as previously described^49,50^. Primary duck embryo fibroblasts (DEF) were derived from embryonated Pekin duck eggs^42^. Both cells were grown in Dulbecco’s modified Eagle’s media (DMEM) supplemented with 10% fetal bovine serum (FBS) and 1% Penicillin–Streptomycin-Amphotericin B (PSA) at 37 °C and 10% CO2.

### Virus propagation and inoculum preparation

Confluent CCL-141 in 100–150 mm dishes (approximately 10^7^ cells/dish) were infected with 10^3^ to 10^4^ focus forming units (FFU) of ABBV-1 (GenBank number: MK966418), and routinely passaged until approximately 100 dishes of persistently infected cells were produced. To release ABBV-1 from cells, growth medium was replaced with 5–10 mL of deionized sterile water for 30 min (osmotic shock), followed by one cycle of freeze/thaw at − 80 °C (ultra-cold freezer). From each dish, cell debris were then dislodged with a cell scraper, the cells/water suspension was pooled, diluted with 10X phosphate-buffered saline (PBS) to reach 1X concentration, vortexed vigorously for 30 s, and then centrifuged for 10 min at 3000 g. The supernatant was collected and incubated overnight at 4 °C with a PEG-8000 (Thermo Fisher) solution (30% w/v) at a 2:1 ratio (supernatant : PEG), and then centrifuged for 15 min at 3200 g. The pellet was finally resuspended in PBS with 1% FBS, for a final ratio of supernatant to final resuspended volume of approximately 100:1. The concentrated virus stock was stored at − 80 °C. A control inoculum was produced in the same way, using CCL-141 not infected with ABBV-1.

### Immunofluorescence and titration of virus stock

The virus stock was titered in CCL-141 cells by limiting dilutions in 96-well plates. Positive wells were detected by immunofluorescence (IFA) using a rabbit monospecific antibody against the N-terminus of the ABBV-1 N protein, as described elsewhere^42,50^. The titers were calculated according to the Spearman-Karber formula as tissue culture infectious dose 50%/mL (TCID_50_/mL), and converted to FFU/mL by multiplying by 0.6951^51^.

### Birds and experimental design

A graphical representation of the experimental layout is provided in Supplementary Figure S1. A total of 174 one-day-old Muscovy ducks (*Cairina moschata*) was purchased from a local hatchery (Webfoot Hatchery, Elora, ON, Canada). The experiment was designed to include 160 birds, with additional birds purchased to account for unexpected mortality (~ 9% attrition rate). Upon arrival, ducklings were randomly divided into 4 rooms (at least 40 birds/room) at the University of Guelph Central Animal Facility Research Isolation Unit (Guelph, ON, Canada), and immediately neck tagged for single bird identification. Ducklings were housed under negative pressure, placed on the floor with 12 h of light–dark, and provided food and water ad libitum. Infrared lamps at floor level were provided until 3 weeks of age.

Approximately 24 h after being received, ducklings were inoculated with ABBV-1 by 1 of 4 routes, each corresponding to a single room: intracranial (IC, 50 μL injected into the subdural space of the right cerebral hemisphere, corresponding to 10^5.65^ FFU/bird), intramuscular (IM, 100 μL injected into the right gastrocnemius muscle, 10^5.95^ FFU/bird), or oral (PO, 100 μL delivered into the oral cavity, 10^5.95^ FFU/bird). Control birds (CO) were sham-inoculated by all three routes using cell lysate from non-infected cells (see inoculum preparation). Intracranial injection was conducted by the same methodology employed for the intracerebral pathogenicity index (ICPI), which is used to test the virulence of avian orthoavulavirus-1 in chickens^52^. Birds were monitored daily, and those that died unexpectedly were sampled as described below. Ten birds at 1, 4, and 8 wpi were randomly selected from each inoculation group, swabbed (oral and cloacal), bled for serum, and euthanized. At 12 wpi, due to the additional birds included in each room, a range of 11–15 birds (total 48) were processed in a similar way.

A complete postmortem analysis was done on all euthanized birds. From each group at each time point, 3 birds were selected for detailed pathology assessment, which entailed collection of over 20 tissues for histopathology (see below); from the remaining birds (range 7–12), 4 tissues only (brain, spinal cord, kidney, and proventriculus) were sampled for both RNA extraction and histopathology. Gonads were collected exclusively from birds at 12 wpi, and used for both RNA extraction and histopathology (Supplementary Figure S1). Sex distribution, as determined at necropsy, was as follows: 18 males, 26 females, and 2 undetermined in the IC group (n = 46); 20 males, 20 females, and 2 undetermined in the IM group (n = 42); 17 males, 24 females, and 1 undetermined in the PO group (n = 42); and 18 males, 23 females, and 1 undetermined in the CO group (n = 42).

Birds at 1 wpi were euthanized by carbon dioxide (CO2) inhalation, after being anesthetized with isoflurane in a 7 L vented induction chamber (VetEquip). Older birds were euthanized by intravenous pentobarbital overdose (100 mg/kg). At 8 and 12 wpi, additional sedation was administered with a mixture of ketamine (30 mg/kg) and dexmedetomidine (0.3 mg/kg) before euthanasia.

Experimental procedures and animal use were approved by the University of Guelph Animal Care and Use Committee (Animal Utilization Protocol 3978). All methods were carried out in accordance with the approved protocol and relevant regulations, and comply with the ARRIVE guidelines.

### Histopathology

From the birds undergoing detailed pathology assessment, the following tissues were harvested: brain, spinal cord (3 segments, cervical, thoracic, lumbar), ischiatic nerves, brachial plexuses, kidneys, gonads, proventriculus, ventriculus, heart, pancreas, adrenal glands, thyroid/parathyroid glands, small intestine (at the level of the Merkel’s diverticulum), colon, lung, liver, spleen, trachea, esophagus, bursa of Fabricius, thymus, and the right gastrocnemius muscle (site of inoculation, only for IM and CO groups). Tissues were fixed in 10% neutral buffered formalin for 48–72 h and then transferred to 70% ethanol until processing. Samples of spinal cord at all time points and brain at 1 wpi were collected with intact vertebrae and skull, respectively, to prevent sampling artifact. In these cases, the tissues were decalcified for 24–48 h after fixation using Cal-Ex II Fixative Decalcifier (Fisher Scientific) prior to trimming. In older birds, the brain was removed from the skull before fixation. For each bird, a coronal section of the cerebrum, optic lobe, brainstem, and cerebellum, and two transverse sections of each segment of the spinal cord were obtained. All remaining tissues were trimmed routinely.

The brain, spinal cord, kidney, proventriculus, and ventriculus from the birds sampled for RNA extraction in the IC and IM groups at 4, 8, and 12 wpi (an additional 48 birds) were also processed for histopathology, to increase the granularity of the scoring (Supplementary Figure S1). These extra birds were chosen based on the detailed pathology assessment of the initial 48 birds, which showed no lesions in the PO and CO groups at any time point. For these birds, only opportunistic samples of the brain could be evaluated as approximately half the tissue was collected for RNA extraction. After trimming, tissues were embedded in paraffin and routinely processed for hematoxylin and eosin (HE).

### Immunohistochemistry

IHC for ABBV-1 was carried out from the tissues of 7 and 1 birds, which were sampled from the IC and CO groups at 12 wpi, respectively. The aim of IHC was to document virus tropism in birds with successful establishment of persistent infection, therefore, the IC group at 12 wpi was chosen as it showed the broadest tissue distribution and highest magnitude of ABBV-1 RNA copies by RT-qPCR.

Immunohistochemistry was performed using a rabbit monospecific antibody against the ABBV-1 N protein (the same used for IFA; 1:6,000 dilution) and visualizing the reaction with Nova Red chromogen (Vector Laboratories), as previously described^42^. A formalin-fixed, paraffin-embedded brain from a Canada goose (*Branta canadensis*) naturally infected with ABBV was used as a positive control^9^. For negative reagent controls, non-immune rabbit serum was used instead of the primary antibody.

Presence of T cells (CD3, rabbit polyclonal antibody raised against the human homologue, Dako) and B cells (Pax5, mouse monoclonal antibody raised against the human homologue, clone 24, BD Biosciences) was determined on the brains from the 3 IC birds selected for detailed pathology assessment at 12 wpi, as previously described^53^. All IHC was conducted by the Animal Health Laboratory (Guelph, ON, Canada).

### Semi-quantitative scoring of nervous lesions and immunohistochemical reactivity

To evaluate difference of inflammation intensity between segments of the central nervous system, the brains and spinal cords from all IC and IM birds at 4, 8, and 12 wpi were scored by one member of the investigative team (M.I.), in a non-blind fashion. Grading was conducted for these two groups only because initial detailed pathology assessment showed no lesions in the CO and PO birds, and in those sampled at 1 wpi across all groups. A bird was considered positive for ABBV-consistent histopathology if lymphocytic-predominant inflammation was appreciated in any segment of the central, peripheral or autonomic nervous tissue.

A semi-quantitative scoring system was developed to quantify the severity of mononuclear inflammation in the central nervous system (Table 5), by recording the thickness of the lymphocytic perivascular cuffs (intensity score) and the number of vessels affected (distribution score). In the brain, lesions were averaged from up to 10, randomly selected 100X fields for each brain area (i.e., cerebrum, cerebellum, brainstem, optic lobe). In the spinal cord, scoring was done for each transverse section of the spinal cord in its entirety; if more than one transverse section was available (range 1–7), the average was recorded. A sub-score was defined as the compiled intensity and distribution scores for each area of the brain or the spinal cord, ranging from 0 to 6. For the entire brain, a score was calculated by adding the sub-scores and dividing the total by the number of available brain areas. Additional lesions of the nervous system (meningitis, peripheral neuritis, and gliosis) were included in a nominal tally.

**Table 5:** Semi-quantitative scoring of inflammation and immunohistochemical reactivity for aquatic bird bornavirus nucleoprotein in the brain and spinal cord of Muscovy ducks experimentally infected with ABBV-1.

Score	Inflammation^a^		Immunohistochemistry^b^
	Intensity	Distribution	Distribution
+ 0	No PVCs^c^ observed	No PVCs observed	No reactivity observed
+ 1	PVCs are 1–2 layers wide	1–10 vessels per 100X field are affected	1–7 positive cells per 400X field
+ 2	PVCs are 3–4 layers wide	11–20 vessels per 100X field are affected	8–14 positive cells per 400X field
+ 3	PVCs are > 5 layers wide	> 21 vessels per 100X field are affected	> 15 positive cells per 400X field
	Sub-score^d^ = intensity score + distribution score (range, 0–6)		Sub-score = distribution score (range, 0–3)
	BRAIN SCORE = (sum of sub-scores from each available anatomical area of brain)/number of areas (range, 0–6)		FINAL SCORE (brain) = sum of sub-score from each anatomical segment of brain (range, 0–12)

^a^The inflammation score accounts for both intensity and distribution.

^b^The immunohistochemistry score accounts for distribution only.

^c^PVCs = perivascular cuffs.

^d^Sub-scores are defined as scores for each segment of the central nervous system (cerebrum, optic lobe, brainstem, cerebellum, spinal cord).

For IHC, presence/absence of ABBV immunoreactivity was tallied for all organs of the tested birds. Semi-quantitative assessment of IHC reactivity was assessed by counting the number of positive cells averaged in up to 10, randomly selected 400X fields for each brain area, or all the complete transverse sections of spinal cord (Table 5). Only cells with intranuclear or intranuclear and cytoplasmic immunolabeling were considered positive.

### Quantification of ABBV-1 RNA by RT-qPCR

Samples (choanal and cloacal swabs, brain, lumbar spinal cord, proventriculus, kidneys, and gonads) were collected into sterile screw cap tubes containing 1.0 mL of preserving solution (20 mM ethylenediaminetetraacetic acid [EDTA], 25 mM sodium citrate, and 70% (w/v) ammonium sulfate with a pH of 5.2), and frozen at − 80 °C until RNA extraction. Total RNA was extracted from 300 mg of tissue or, for swabs, 300 μL of preserving solution using E.Z.N.A RNA Kit II (Omega Bio-Tek), following the manufacturer’s protocol. Purified RNA was reverse transcribed and amplified using a Luna Universal Probe one-step RT-qPCR kit (NEB) with primers and probes targeting the ABBV-1 N gene (forward, 5′-ATG CAC TTG CAC TCT TAG AC-3′; reverse, 5′-TCC CCA TAA AAC CTC CCA AC-3ʹ; probe, 5′-6-FAM-CCC TGC CCG CAG AGA GAA ATT CCA T-BHQ-3′). The cycling conditions were as follows: 55 °C for 10 min reverse transcription; 95 °C for 1 min initial denaturation, and 40 cycles of 95 °C for 10 s denaturation and 60 °C combined annealing and extension. Samples with cycle threshold (Ct) less than 35 were considered positive.

Virus RNA copies were determined based on a standard curve produced using ten-fold dilutions (3 × 10^8^ to 3 × 10^1^ copies/reaction) of a gene cassette containing a 500 bp fragment of the ABBV-1 N gene (Integrated DNA Technologies), and run in parallel with each plate. The final output of the PCR was reported as RNA copies per 150 ng of total extracted RNA (tissues), or per 84 µL of swab fluid.

### Serology

The sera from all birds at 4, 8 and 12 wpi from the IC (n = 10, 10, 15), IM (n = 10, 10, 11), PO (n = 10, 10, 11) and CO (n = 10, 10, 11) groups were tested for seroconversion against ABBV-1 using an ELISA (enzyme-linked immunosorbent assay) test. Birds at 4 wpi were selected as encephalitis was highest at this time point; birds at 8 and 12 wpi (later timepoints) were selected as expected to have the highest degree of seroconversion in chronically infected birds. Briefly, wells of a non-treated 96-well microtiter ELISA plates (ThermoFisher) were coated with full-length recombinant ABBV-1 N protein (Biomatik) at 25 ng/well, diluted in bicarbonate/carbonate coating buffer (50 mM, pH 9.5) for 2 h at 37 °C. After excess antigen was removed by five washes [3 washes with PBS-T (0.05% v/v Tween-20 in PBS) followed by 2 washes with ddH2O], wells were blocked with 5% fish gelatin in PBS overnight at 4 °C. All subsequent wash steps are the same. After five washes post-blocking, either 50 uL of test sera (1:200 diluted in PBS-T with 1% fish gelatin) or 50 uL of monospecific rabbit antibody against the ABBV-1 N protein (1:500 to 1:64,000 dilution series in PBS-T with 1% fish gelatin) was added in triplicate wells. Before use, sera were clarified a second time by centrifugation. The monospecific rabbit antibody against the ABBV-1 N protein, the same employed for IFA and IHC, was used as a positive control for the ELISA reaction. Plates were incubated for 2 h at 37 °C, washed five times, and incubated with 50 uL of a goat anti-avian IgG-heavy and light chain HRP conjugated secondary antibody (1:10,000) (Cedarlane), or goat anti-rabbit HRP conjugated secondary antibody (1:2,000) (ThermoFisher) for 2 h at 37 °C. Both secondary antibodies were diluted in PBS-T with 1% fish gelatin. After five washes, wells were developed by adding 50 uL/well of Pierce 1-Step Ultra TMB-ELISA Substrate Solution (ThermoFisher) for 10 min, and the reaction was stopped by adding 50 uL of 2 N Sulfuric acid. Plates were read at 450 nm using an EnSpire multimode plate reader (Perkin Elmer).

A calibrator, made by mixing equal volumes (100 uL) of 2 CO birds from each time point (total, 6), was run in triplicate in each plate. The raw OD values of each plate were divided by its calibrator (fold change values), and then multiplied by the average of all the calibrators across all plates (normalized values). The normalized OD of all the CO birds across the three time points, plus three-time the standard deviation was used as the cut-off value to determine positive sera.

To test the immunogenicity of the recombinant protein used to coat the ELISA plates, the protein was immunoblotted in parallel with cell lysates of ABBV-1 infected cells, using a cohort of sera, as previously described^50^ (see supplementary material). Specifically, from each experimental group at 12 wpi, one serum sample with highest and one with lowest OD were tested in immunoblots. The recombinant protein was similarly immunoblotted to verify reactivity with the monospecific rabbit antibody used in IFA and IHC.

### Virus isolation

To evaluate if the RNA detected by RT-qPCR corresponded to infectious virus, isolation was conducted from the viable brains of all birds in the IC (n = 11) and IM (n = 8) groups at 12 wpi, as well as 4 CO birds at the same time point. Virus isolation was not carried out for the PO birds, as these were consistently negative for virus RNA by RT-qPCR at all time points. Virus isolation was also attempted from the kidneys of two IC and one CO birds at 12 wpi. Two representative birds of the IC group were chosen, as in these birds, renal tissues showed presence of ABBV-1 RNA by RT-qPCR, but no IHC reactivity.

For each sample, a small piece of tissue was rinsed in PBS, added to a tube containing 1 mL of 10% FBS/DMEM, and homogenized using the Precellys 24 homogenizer (Bertin Instruments) for 40 s (2—20 s bursts at 5000 rpm with 5 s pause between bursts). 500 μL of tissue homogenates were layered on top of confluent DEF monolayers in 12-well plates containing 500 μL of 10% FBS/DMEM per well. The next day, the cells were washed three times in PBS and medium was replaced. Cells were then routinely passaged up to four times (25 days post-inoculation), when presence of ABBV-1 in cells was detected by IFA.

### Statistical analysis

The average magnitude of virus RNA copies in tissues and swabs, as determined by RT-qPCR, was compared between different time points and inoculation routes using a two-way ANOVA test (variables: inoculation route and time point), with multiple comparisons between groups (Tukey’s test). Group differences in the semi-quantitative histology, immunohistochemistry scores, and the magnitude of seroconversion were compared using the Kruskal–Wallis test followed by multiple comparisons using the Benjamini, Krieger, and Yekutieli procedure for false discovery rate (q < 0.05).

Univariable regression models were built using the brain and spinal cord pathology scores in the IC and IM groups as dependent variables, and time post infection (4, 8, 12 wpi), and virus RNA copies in select organs (brain, spinal cord, and proventriculus) as explanatory variables, using a relaxed p value (*P* ≤ 0.2). Spearman’s correlation was further used to visualize association between variables in univariable analysis. Only significant variables were then included into a multivariable model. Virus RNA concentration was expressed as log 10 copy numbers per 150 ng of total RNA, and zero values were transformed to 0.00001. For each model, 26 observations were present. While the Q-Q plots were approximately normally distributed, the homoscedasticity assumption was not fully met, likely due to the relatively small sample size. Nonetheless, our models had merit and were considered in the analysis^54^.

Statistical analysis was carried out using Stata, version 14.0 (Stata Corporation, College Station, Texas, USA) for comparison of proportions and regression analysis, or GraphPad Prism for iOS, version 9 (GraphPad Software, La Jolla, USA) for the other tests. Significance was set at *P* < 0.05.

### Ethics approval

Animal use and experimental procedures involving animals were approved by the University of Guelph Animal Care and Use Committee (Animal Utilization Protocol #3978).

## Supplementary Information

Supplementary Information.

## Data Availability

Any additional data is submitted as Supplementary Material.
