# Supplementary material for: Experimental infection of aquatic bird bornavirus in Muscovy ducks
Source: Sci Rep. 2022 Sep 30;12:16398. doi: 10.1038/s41598-022-20418-x (PMC9525603; doi:10.1038/s41598-022-20418-x)

SUPPLEMENTARY MATERIAL:

Experimental Infection of Aquatic Bird Bornavirus in Muscovy Ducks

Melanie Iverson[1], Alexander Leacy[1], Phuc H. Pham[1], Sunoh Che[1], Emily Brouwer[2], Eva Nagy[1], Brandon N. Lillie[1], Leonardo Susta[1]*

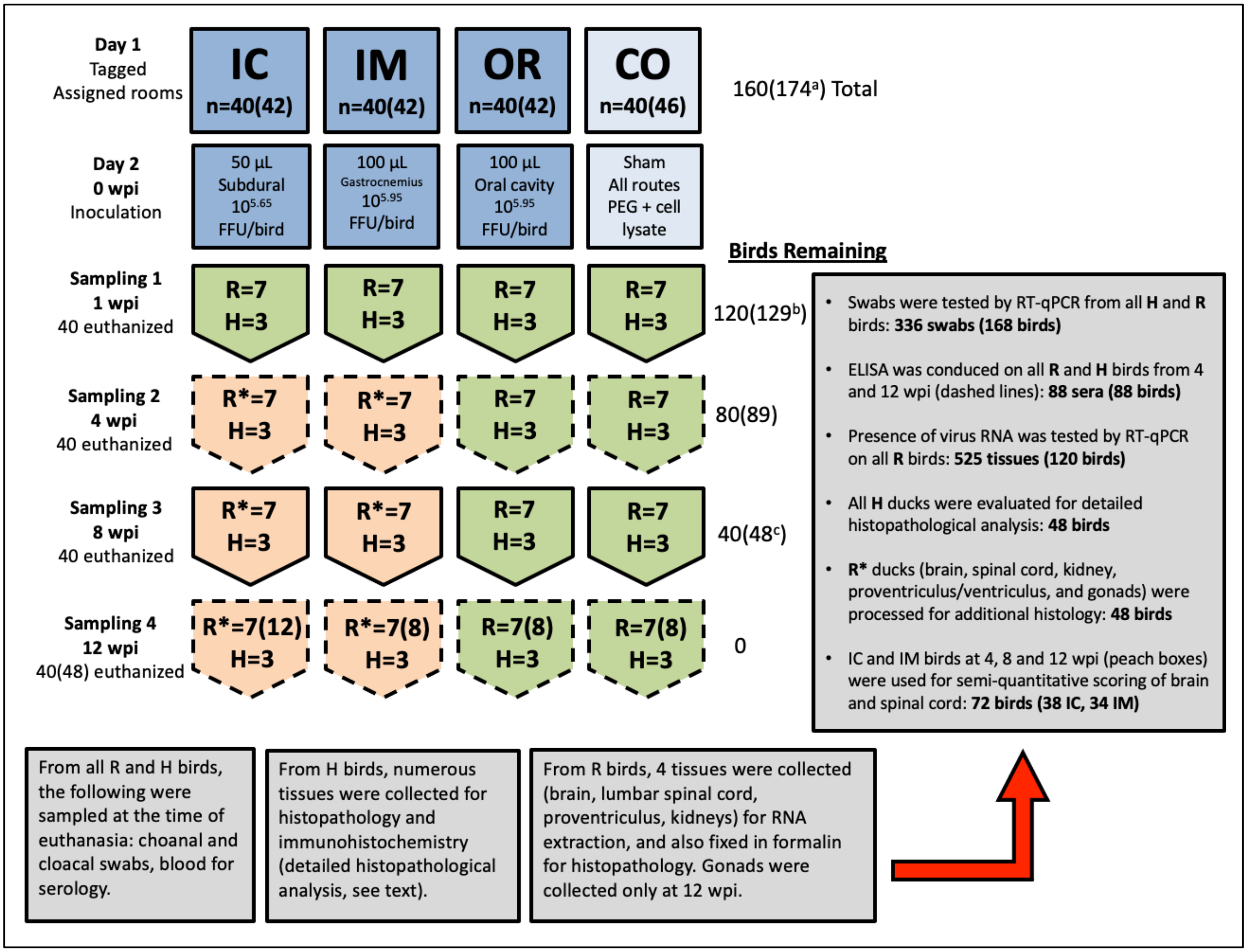

Supplementary Figure S1. Schematic representation of the experimental plan for infection of Muscovy ducks with ABBV-1. Reported are the virus inoculum, the number of birds in each experimental groups (IC, intracranial; IM, intramuscular; PO, oral; CO, control), euthanasia schedule, and the sampling protocol for downstream testing. "H", indicates birds that were sampled for detailed pathology assessment; "R", indicates birds sampled for detection of virus RNA by RT-qPCR. "R*", indicated birds

in the R cohort that were further processed for histology. Boxes in peach color indicates birds used for semi-quantitative histopathological scoring (as no lesions were identified in the other groups). Birds in boxes with dashed lines were used for serology by ELISA. Numbers in parenthesis indicate the actual number of ducks used and remaining at each time point, based on unexpected deaths and extra birds. See text for detailed description. [a]Two ducklings were euthanized at two days of age, prior to inoculation, to be used as early control birds. [b]Two ducklings died immediately after inoculation and 1 died unexpectedly at 3 days post infection (dpi). [c]One duckling died unexpectedly at 46 dpi.

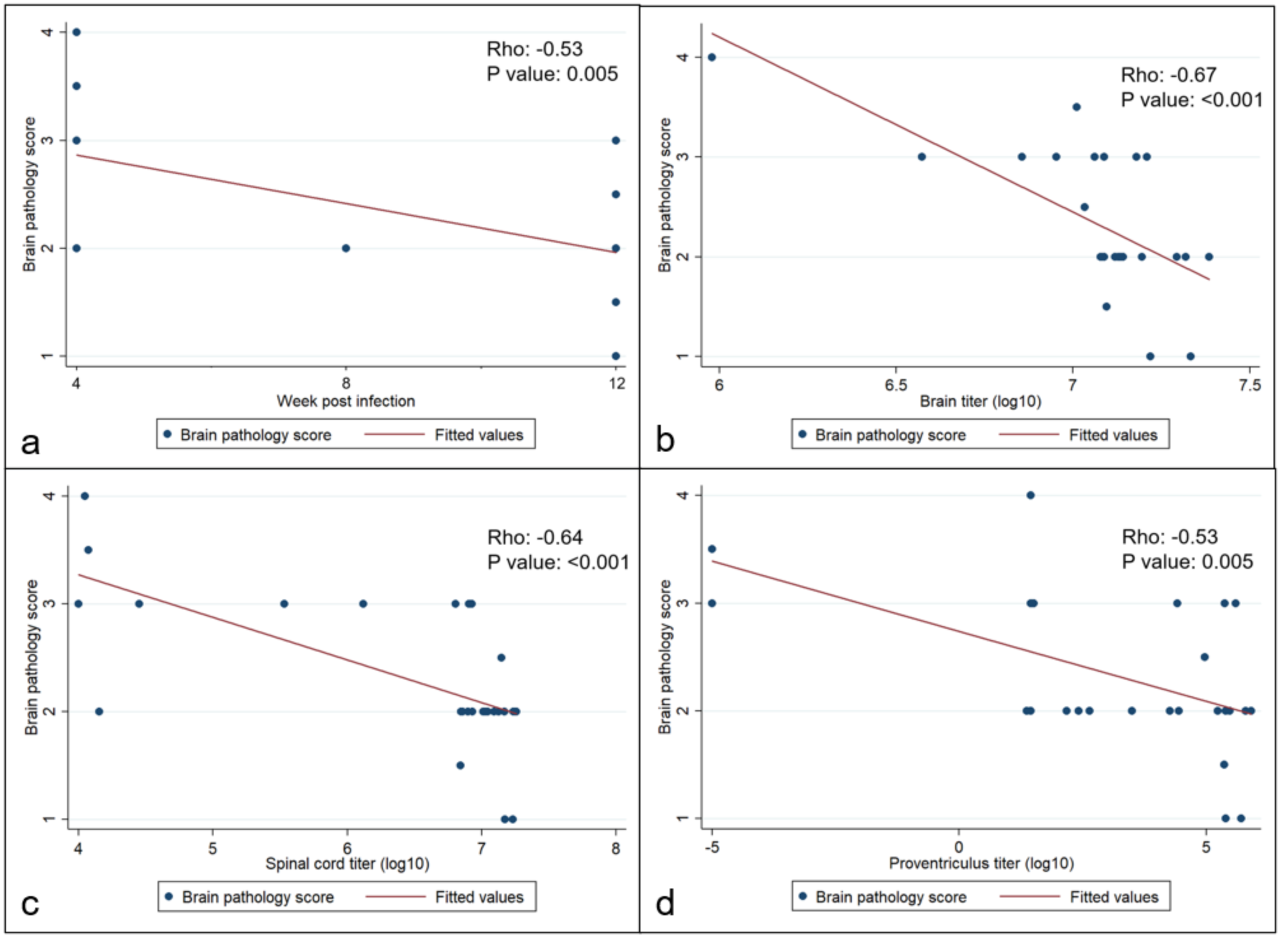

Supplementary Figure S2. Scatter plots showing the association between independent variables [week post infection (4, 8, 12 wpi), and virus RNA copies (titer) in brain, spinal cord, and proventriculus] and dependent variable (brain pathology score). Spearman's correlation coefficients (Rho), as well as significance are reported on the top right of each panel. Upon multivariable regression analysis, only the correlation between brain pathology score and brain titer (virus RNA copies) was significant (panel b).

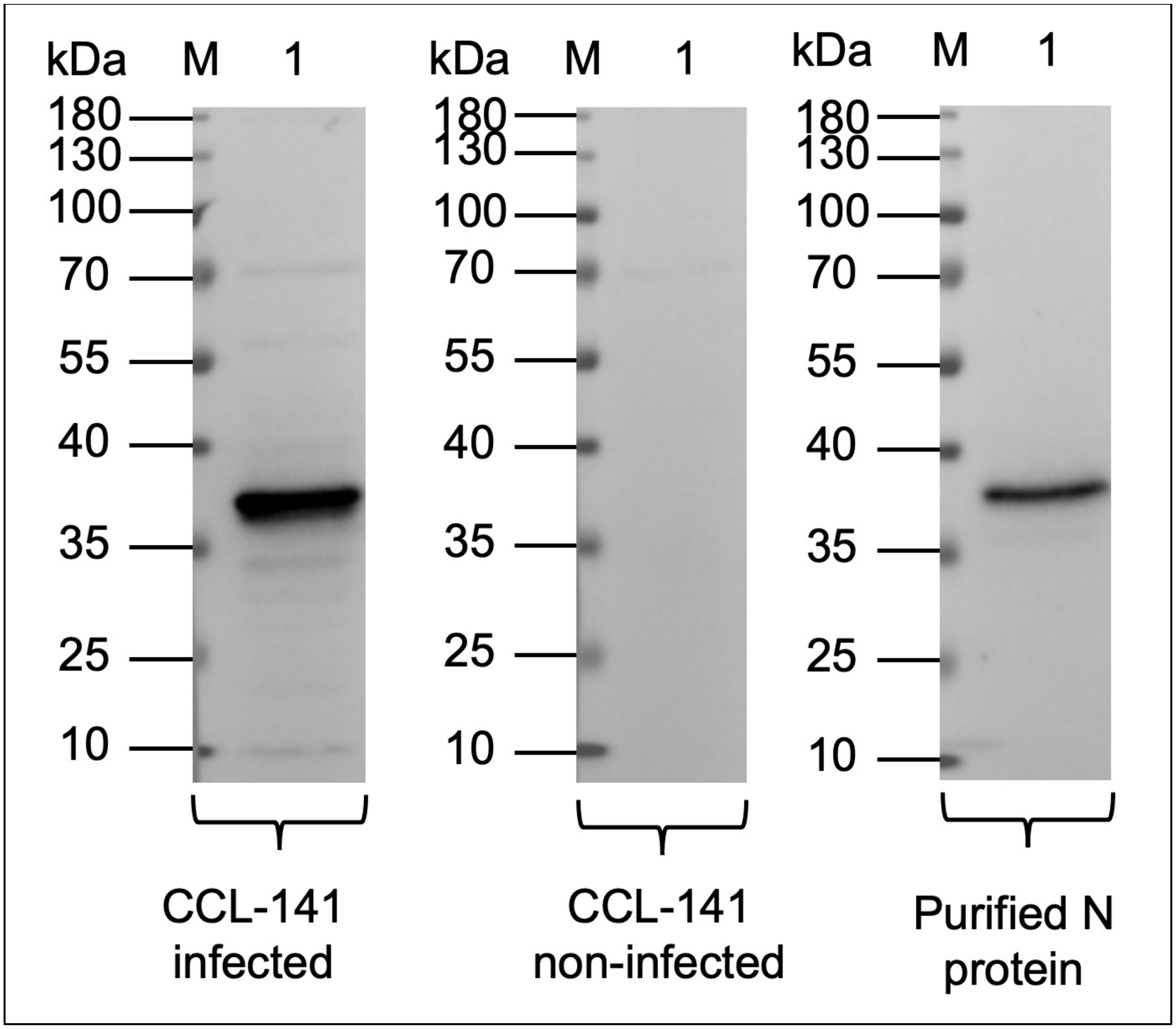

Supplementary Figure S3. Immunoblots using a monospecific rabbit antibody against the N terminus of the ABBV-1 N protein. The antibody detects a protein band between 40 and 35KDa (consistent with ABBV-1 N protein) in blots loaded with both the cell lysates from ABBV-1 infected cell (immortalized duck fibroblasts) and the recombinant N protein. The blot with cell lysate from non-infected cells shows no band of interest.

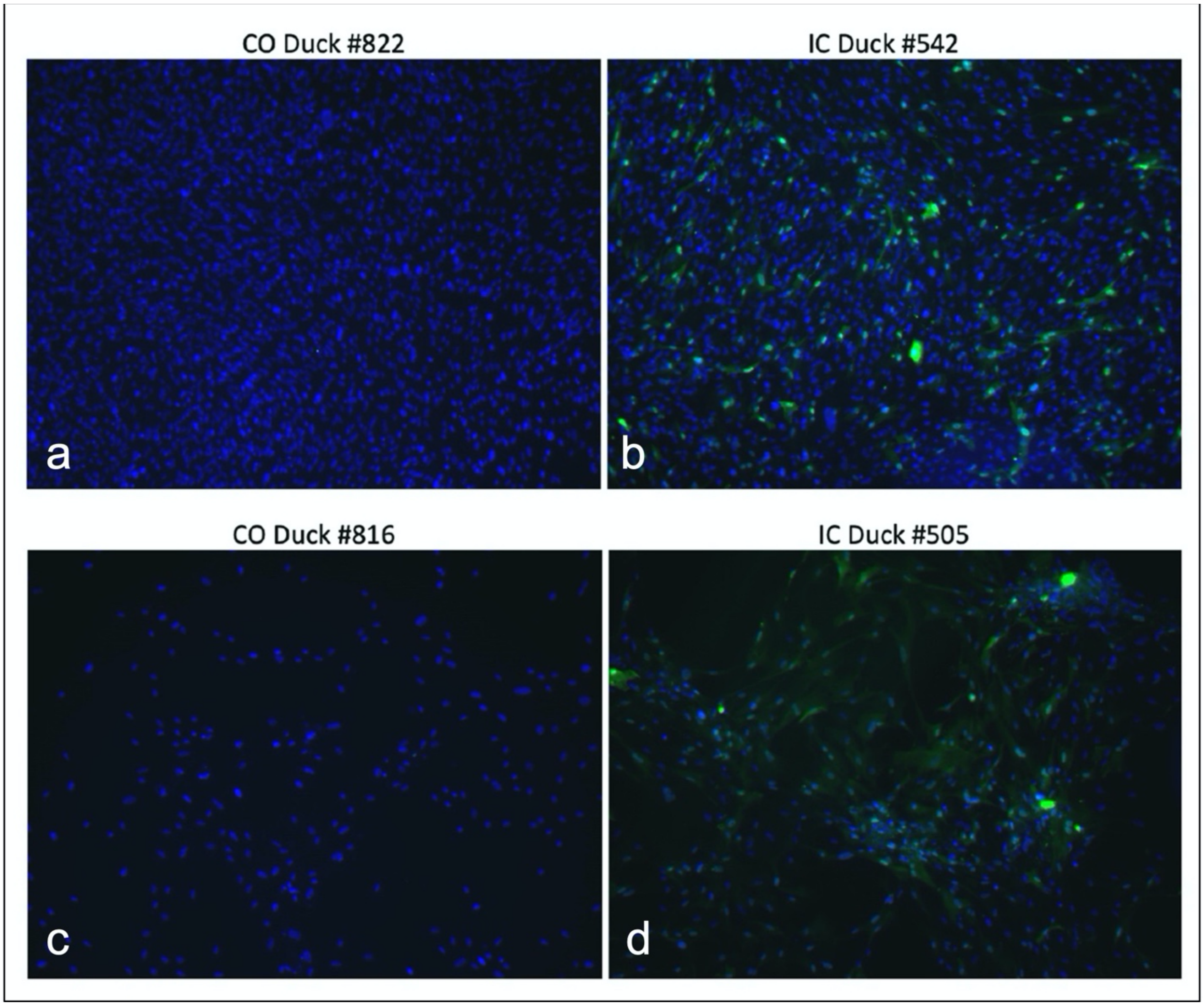

Supplementary Figure S4. (**a, b**) Isolation of ABBV-1 from the brain of an experimentally infected Muscovy duck at 12 wpi. Immunofluorescence assay for ABBV-1 N antigen in control (CO) duck #822 and intracranial (IC) duck #542 at passage 3. The brain from the IC duck yielded ABBV-1 in CCl-141 after three passages, as shown by nuclear signal in scattered cells. No signal is appreciated in the brain of the CO duck. (**c, d**) Isolation of ABBV-1 from the kidney of an experimentally infected Muscovy duck at 12 wpi. Immunofluorescence assay for ABBV-1 N antigen in control (CO) duck #816 and intracranial (IC) duck #505 at passage 3. The kidney from the IC duck yielded ABBV-1 in CCl-141 after three passages, as shown by nuclear signal in scattered cells. No signal is appreciated in the kidney of the CO duck.

Supplementary Materials and Methods for Western Blot. Fifty (50) ng of purified N protein or 20 µg of cell lysate from ABBV-1-infected and non-infected CCL-141 cells were resolved on a 12 % SDS-PAGE (120 V for 1.5 h) and transferred to a PVDF membrane at 25 V for 30 min (semi-dry transfer; Bio Rad). Membranes were blocked with either 5 % skim milk in PBS-T at 4 °C overnight. Blots were either incubated with duck sera (1:1000 dilution, for **Figure 6b**) or rabbit monospecific antibody (1:4000, for **Supplementary Figure S3**) overnight at 4 °C, and then incubated 1 h at room temperature with a secondary goat anti-avian IgY antibody (1:20000; A140-110P-Bethyl Laboratories Inc., Cedarlane) or anti-rabbit IgG (1:2000; Thermofisher), both conjugated with horseradish peroxidase (HRP). Signal was detected by incubating with the SuperSignal™ West Pico PLUS Chemiluminescent Substrate (ThermoFisher) for at least 5 min before band detection using a ChemiDoc MP Imaging System and Image Lab 6.0.1. software (Bio Rad).

Full membranes for the immunoblots shown in **Figure 6** and **Supplementary Figure S3** are shown below

Full membranes for figure 6

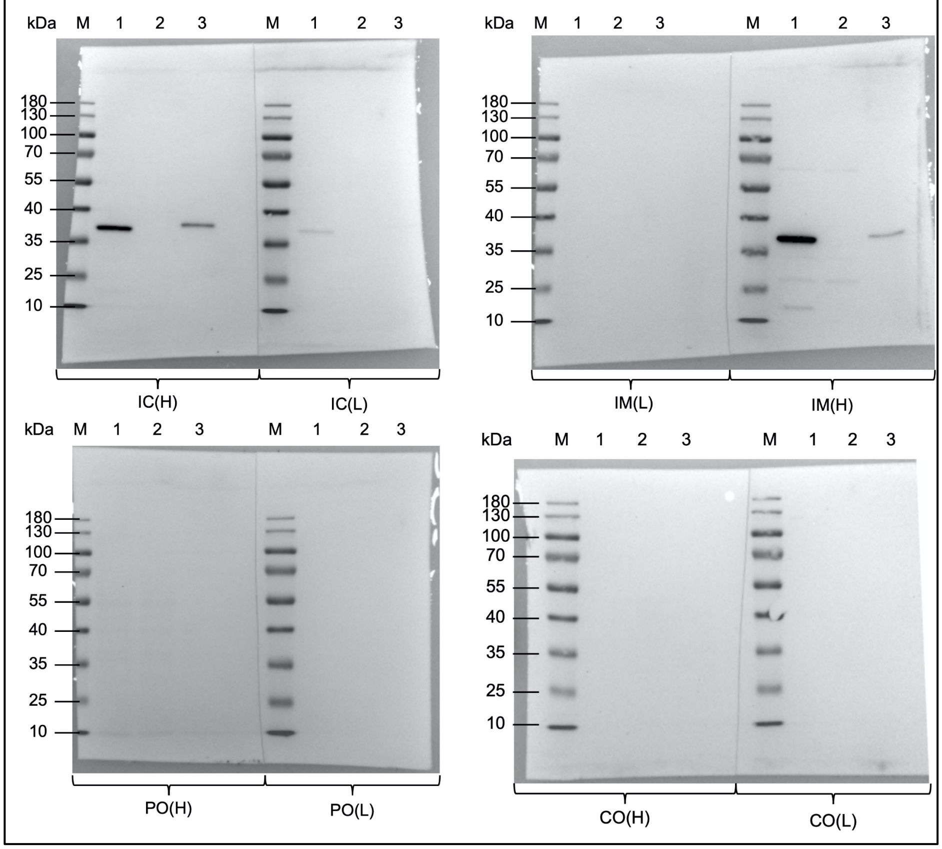

Full membranes for Figure S3

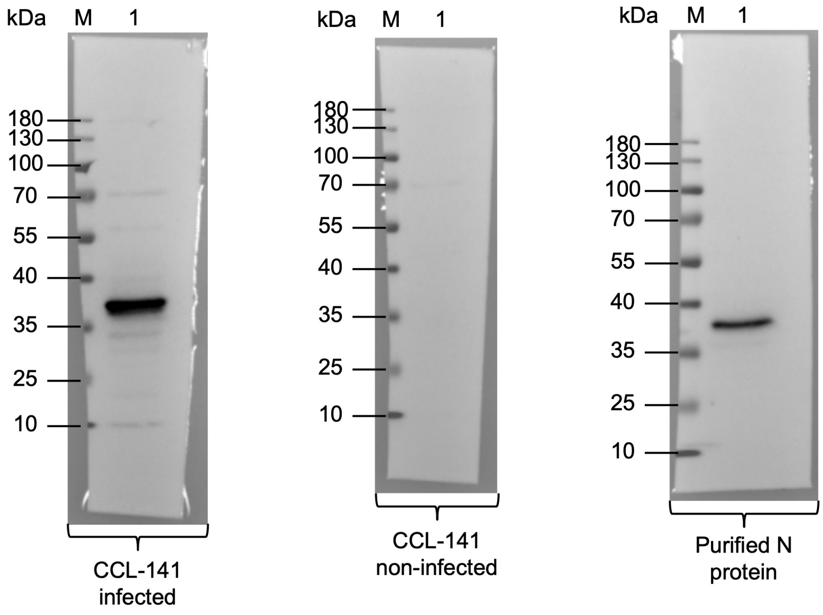

Supplement: Supplementary file 1 — Supplementary Information. [file 41598_2022_20418_MOESM1_ESM.pdf]
